# Study on the pro-inflammatory injury of neutrophil extracellular traps in gouty nephropathy

**DOI:** 10.3389/fimmu.2026.1830649

**Published:** 2026-04-29

**Authors:** Shuliang Pu, Niqin Xiao, Dongyun Li, Xin Cai, Jing Xie, Zhaohu Xie, Danxia Wei

**Affiliations:** 1 The Third Affiliated Hospital of Yunnan University of Chinese Medicine, Kunming, Yunnan, China; 2 Yunnan University of Chinese Medicine, Kunming, Yunnan, China

**Keywords:** Gouty nephropathy, inflammation, neutrophil extracellular traps, renal injury, therapy

## Abstract

Gouty nephropathy is a common complication of gout, which is characterized pathologically by the abnormal deposition of urate crystals in the kidneys. This deposition can directly trigger local inflammatory responses and lead to renal tissue damage. Neutrophil extracellular traps (NETs), as a unique neutrophil-derived immune defense mechanism, play a key pro-inflammatory role in gouty nephropathy. Under conditions of long-term hyperuricemia, Monosodium urate (MSU) crystals can activate related inflammatory signaling pathways, promoting inflammatory cell infiltration, the release of pro-inflammatory factors, and the formation of NETs, thereby exacerbating inflammatory responses and fibrosis in hyperuricemic nephropathy. This review summarizes the formation and pro-inflammatory injury mechanisms of NETs in gouty nephropathy and discusses the potential and challenges of targeting NETs as a novel therapeutic strategy for gout-related kidney disease.

## 1 Introduction

Gouty nephropathy is one of the common complications of gout. Long-term hyperuricemia leads to the deposition of Monosodium urate (MSU) crystals in renal tissues, which can trigger chronic inflammation, fibrosis, and a decline in renal function (1). However, its pathogenesis is not yet fully understood. Current urate-lowering drugs mainly include inhibitors of uric acid synthesis, uricosuric agents, and uricolytic drugs, but these medications are associated with specific adverse effects and clinical limitations. Uric acid synthesis inhibitors may induce severe hypersensitivity reactions and increase potential cardiovascular risks, uricosuric drugs can induce hepatotoxicity, and uricolytic agents may also trigger serious hypersensitivity responses (2). Therefore, treatment remains challenging for some patients, highlighting the urgent need for drug interventions and therapeutic approaches based on novel pathological mechanisms.

Neutrophil extracellular traps (NETs), as a key component of the innate immune response, have increasingly attracted attention regarding gout-related inflammation and renal injury (3). In gouty inflammation, activated neutrophils release NETs, which play a significant pro-inflammatory role. Uric acid or urate crystals can serve as stimulatory signals that effectively trigger the formation and release of NETs, amplifying local inflammatory responses and exacerbating renal tissue damage (1, 4). Given the multiple key roles of NETs in the development and progression of gouty nephropathy, NETs can be considered an important therapeutic target.

This article aims to explore potential new strategies for treating gouty nephropathy from the perspective of NETs pro-inflammatory effects. In other renal injury diseases, NETs directly damage renal tubular epithelial cells by releasing toxic substances such as cathepsins and myeloperoxidase, and participate in inflammatory responses and tissue fibrosis, thereby aggravating renal tissue injury (5, 6) This provides a new perspective for studying the mechanisms of gout-related renal damage. Therefore, considering the mechanism by which NETs exacerbate tissue injury, we can explore a new therapeutic direction. In addition to traditional urate-lowering treatments, interventions targeting the pro-inflammatory and injurious effects of NETs may become a potential therapeutic approach. Currently, the application of NETs-targeted therapies in gouty nephropathy remains limited. However, we can draw on NETs targeting strategies used in other diseases and apply them to the study of gouty nephropathy treatment. Specific strategies include targeting the inhibition of NETs formation such as using Peptidylarginine deiminase 4( PAD4)inhibitors, neutralizing toxic components such as histones released by NETs, or promoting the degradation of the DNA mesh structure of NETs such as using deoxyribonuclease I(DNase I) (7–9). These methods provide potential new insights for alleviating renal tissue damage caused by NETs induced by uric acid crystals. Furthermore, traditional indicators of renal function, such as serum creatinine and glomerular filtration rate, are to some extent unable to effectively distinguish whether kidney injury is inflammatory or chronic non-inflammatory damage. Therefore, research on related biomarkers for NETs diagnostic markers also represents another perspective for a deeper understanding of the potential pathophysiological mechanisms of gouty nephropathy.

## 2 Characteristics and formation of neutrophil extracellular traps

NETs are extracellular mesh-like structures released by neutrophils in response to inflammatory stimuli or pathogen infection (10). Their core components consist of decondensed chromatin Deoxyribonucleic acid (DNA) serving as the scaffold, decorated with histones and granular proteins such as Neutrophil elastase (NE) and Myeloperoxidase (MPO) (6, 11–13). Under physiological conditions, the DNA meshwork traps pathogens, limiting their dissemination within the host, while the granular proteinases form a high-concentration “bactericidal zone” that efficiently degrades pathogen virulence factors and directly disrupts their cell membranes, thereby exerting antimicrobial effects (14). However, when this inherently host-protective mechanism becomes dysregulated, it can induce tissue inflammation and injury under pathological conditions (15, 16), extending beyond its aforementioned bactericidal function (4, 17–20). For instance, when MSU crystals deposit in the kidneys, they recruit and activate neutrophils, which can induce Neutrophil extracellular trap formation (NETosis) and release large amounts of NETs (4, 21). Acting as inflammatory mediators, NETs can synergistically damage renal tissue through multiple mechanisms, including cytotoxicity, sustained amplification of inflammatory circuits, complement activation, and microthrombosis. This leads to acute kidney injury and promotes the initiation and progression of renal fibrosis (6, 22–24).

Currently, based on the dependency on Reactive oxygen species (ROS) sources for their formation, the processes of NETs generation primarily include Nicotinamide adenine dinucleotide phosphate (NADPH) oxidase-dependent NETosis, NADPH oxidase-independent NETosis, and mitochondrial NETosis (25–27). Although multiple pathways exist, existing research on gouty nephropathy has mainly focused on the NADPH oxidase-dependent pathway. The roles of these alternative pathways in this disease remain unclear and warrant further investigation. When MSU crystals are recognized by neutrophils, they induce an influx of extracellular calcium, leading to an increase in cytosolic calcium concentration. This subsequently activates Peptidylarginine deiminase 4 (PAD4) and Protein kinase C (PKC). PAD4 catalyzes the citrullination of histones (28), facilitating chromatin decondensation, nuclear membrane rupture, and the intermingling of chromatin with granular proteins to form the meshwork. While the cell remains viable, these components are released via exocytosis to trap or kill pathogens (29–31). Additionally, activated PKC can phosphorylate and activate the NADPH oxidase complex. The activated NADPH oxidase generates a large amount of ROS, which subsequently activates myeloperoxidase and neutrophil proteases. These enzymes translocate to the nucleus, where they further promote chromatin decondensation (32).

## 3 Pathological features and pathogenesis of gouty nephropathy

Microscopic examination of gouty nephropathy reveals substantial deposition of urate crystals in the renal interstitium, surrounded by inflammatory infiltrates of macrophages, lymphocytes, and eosinophils (33). Degenerative changes such as basement membrane thickening and focal segmental sclerosis can occur in the tubular regions. These pathological alterations lead to intrarenal microcirculatory disturbances, a persistent reduction in renal blood flow, a consequent decline in the glomerular filtration rate, gradual loss of nephron function, and eventual progression to end-stage renal failure (34, 35).

The pathogenesis of gouty nephropathy is primarily driven by hyperuricemia-induced kidney damage (36). In the kidneys, approximately 90% of Uric acid (UA) is reabsorbed into the bloodstream. Elevated uric acid levels promote the precipitation of urate crystals in the renal interstitium and within tubules (33). These crystals can directly injure renal tubular epithelial cells (37–39), leading to their atrophy and triggering local tissue inflammatory responses (40, 41). Furthermore, the deposition of urate crystals also contributes to interstitial fibrosis and glomerulosclerosis (42, 43).

When the kidneys are chronically exposed to a hyperuricemic environment, the Nuclear factor kappa-B (NF-κB) signaling pathway serves as a key mediator of inflammatory responses (36, 44–47) and is also involved in oxidative stress. The inflammation and oxidative stress mediated by the NF-κB pathway can exacerbate renal injury. Within the inflammatory response, the NOD-like receptor family, pyrin domain containing 3 (NLRP3) inflammasome (4, 33, 48) and the NF-κB signaling pathway are two critically interconnected core components. Urate crystals can directly activate the NLRP3 inflammasome in macrophages, prompting caspase-1 to cleave pro-Interleukin-1β (pro-IL-1β) and release large amounts of mature IL-1β (21, 49), which subsequently recruits neutrophils to infiltrate the renal tissue. Additionally, upon recognition by renal tubular epithelial cells or infiltrating immune cells, urate crystals can activate the NF-κB signaling pathway via Toll-like receptor 4 (TLR4) or Toll-like receptor 2 (TLR2) (36). Activation of the NF-κB pathway promotes the expression of pro-inflammatory cytokines, chemokines, and adhesion molecules, leading to the extensive recruitment and infiltration of immune cells such as neutrophils into the renal tissue. Concurrently, NF-κB activation provides a necessary condition for the full activation of the NLRP3 inflammasome, for instance, by upregulating the expression of pro-IL-1β, thereby further amplifying the inflammatory response (36). Thus, urate crystals sequentially activate the NLRP3 inflammasome and the NF-κB signaling pathway, forming a potent inflammatory signaling network. This sustained inflammatory reaction promotes the progression of renal fibrosis. Although the above signaling pathways play important roles in gouty nephropathy, they cannot fully explain the pathogenesis of inflammatory gouty nephropathy. The discovery of neutrophil extracellular traps (NETs) may have potential significance and value in improving the explanation of this pathogenesis.

**Figure 1:**
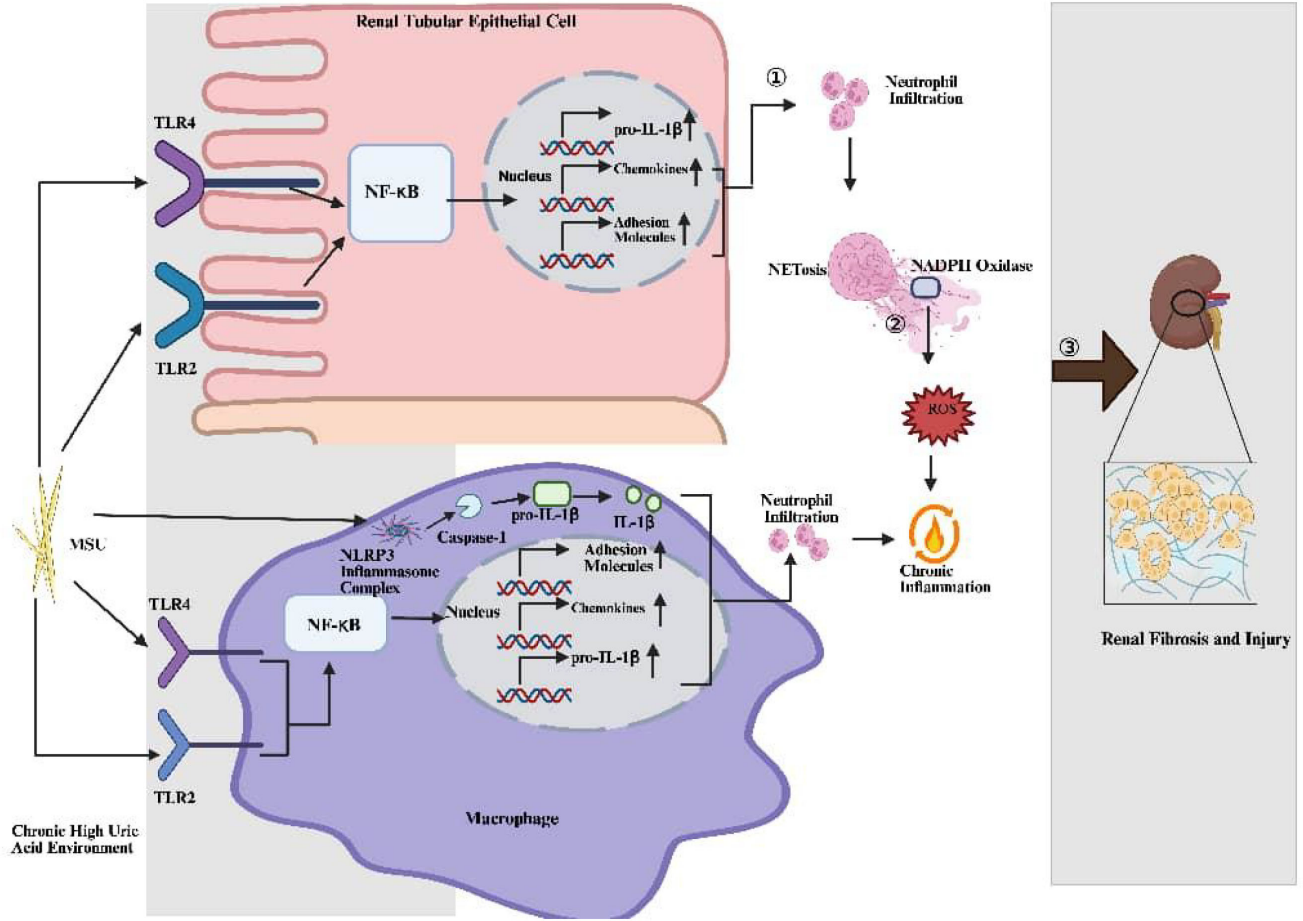
Pathological characteristics and related pathogenesis of gouty nephropathy ① In a hyperuricemic environment, multiple stimuli can activate the NF-κB signaling pathway. This pathway upregulates the expression of pro-inflammatory factors, chemokines, and adhesion molecules, further leading to neutrophil infiltration. ② During NETosis, the activation of NADPH oxidase generates a large amount of ROS. ③ The components of NETs and the excessive production of reactive oxygen species contribute to the development of renal fibrosis, which further results in renal injury.

Recruited and activated neutrophils undergo NETosis and release NETs, during which process, accompanied by the activation of nicotinamide adenine dinucleotide phosphate (NADPH) oxidase (NOX), a massive burst of reactive oxygen species (ROS) is generated (50). These ROS pose a dual threat. Firstly, they can directly cause oxidative damage to renal tubular epithelial cells and glomerular endothelial cells. Secondly, ROS can directly activate the NF-κB pathway, for example by oxidatively modifying the IκB kinase (IKK) complex, leading to the initiation of inflammatory responses (51). Importantly, the activation of the NF-κB pathway can, in turn, upregulate the expression of oxidases like NOX, increasing ROS production, which then feedback to activate NF-κB.In addition, the structure of NETs itself can serve as a new signal, reactivating the NF-κB signaling pathway in macrophages. Therefore, in the context of NETs presence, a persistent “oxidative stress-inflammation” positive feedback loop is established, further contributing to renal injury (**Figure 1**).

## 4 The role of NETs in gouty inflammation

### 4.1 Anti-inflammatory and inflammatory resolving effects: clearance and regulatory functions of NETs

NETs play a role in the resolution of gouty inflammation. Their anti-inflammatory mechanisms include physical clearance and sequestration. When NETs reach a certain density, they can form aggregated neutrophil extracellular traps (aggNETs), which can physically envelop MSU crystals via their DNA meshwork, effectively isolating these inflammatory triggers, limiting crystal dispersion, and promoting their phagocytosis and clearance by macrophages (52). The second mechanism is the degradation of inflammatory mediators. The NET structure contains serine proteases that can degrade inflammatory cytokines and chemokines such as interleukin-8 (IL-8) and C-X-C motif chemokine ligand 1 (CXCL1), thereby blocking further recruitment and activation of neutrophils and negatively regulating the inflammatory response (53). These anti-inflammatory mechanisms of NETs help explain why gout patients may experience a period of spontaneous pain relief despite the persistence of MSU crystals in the body. The repeated accumulation of these crystals within the body leads to the development of gouty nephropathy. Therefore, clinical management should focus on mitigating NET-related pathological products to control disease progression and promote inflammation resolution in patients with gouty nephropathy (54–56). Thus, early attention to NETs can effectively prevent the onset of gouty nephropathy.

### 4.2 Pro-inflammatory effects: initiating and amplifying inflammation and inducing renal tissue injury

In the chronic pathological process of gouty nephropathy, the limited anti-inflammatory function of NETs is insufficient to counteract the vicious cycle triggered by MSU crystal deposition. The pro-inflammatory effects of NETs likely predominate in gouty nephropathy. Upon the deposition of uric acid crystals in renal tissue, urate crystals can activate the NLRP3 inflammasome and caspase-1 pathway in macrophages (21, 57–63), inducing the release of potent pro-inflammatory factors such as IL-1β, which in turn recruits neutrophils to infiltrate the inflammatory site. NETs have multiple pro-inflammatory and injurious effects. Firstly, NETs can directly damage tissues by releasing NE and MPO (53, 64). Second, histones, key components of NETs, act as potent inflammatory mediators by activating Toll-like receptor signaling pathways in macrophages; this subsequently triggers NLRP3 inflammasome activation and IL-1β release, thereby amplifying inflammation and inducing tissue damage (59). Additionally, NETs can promote macrophage polarization towards the pro-inflammatory M1 phenotype by targeting Hexokinase 2 (HK2), upregulating the expression of Cluster of Differentiation 86 (CD86), Cluster of Differentiation 80(CD80), and Inducible nitric oxide synthase (iNOS), further exacerbating local inflammatory responses (57).

**Figure 2:**
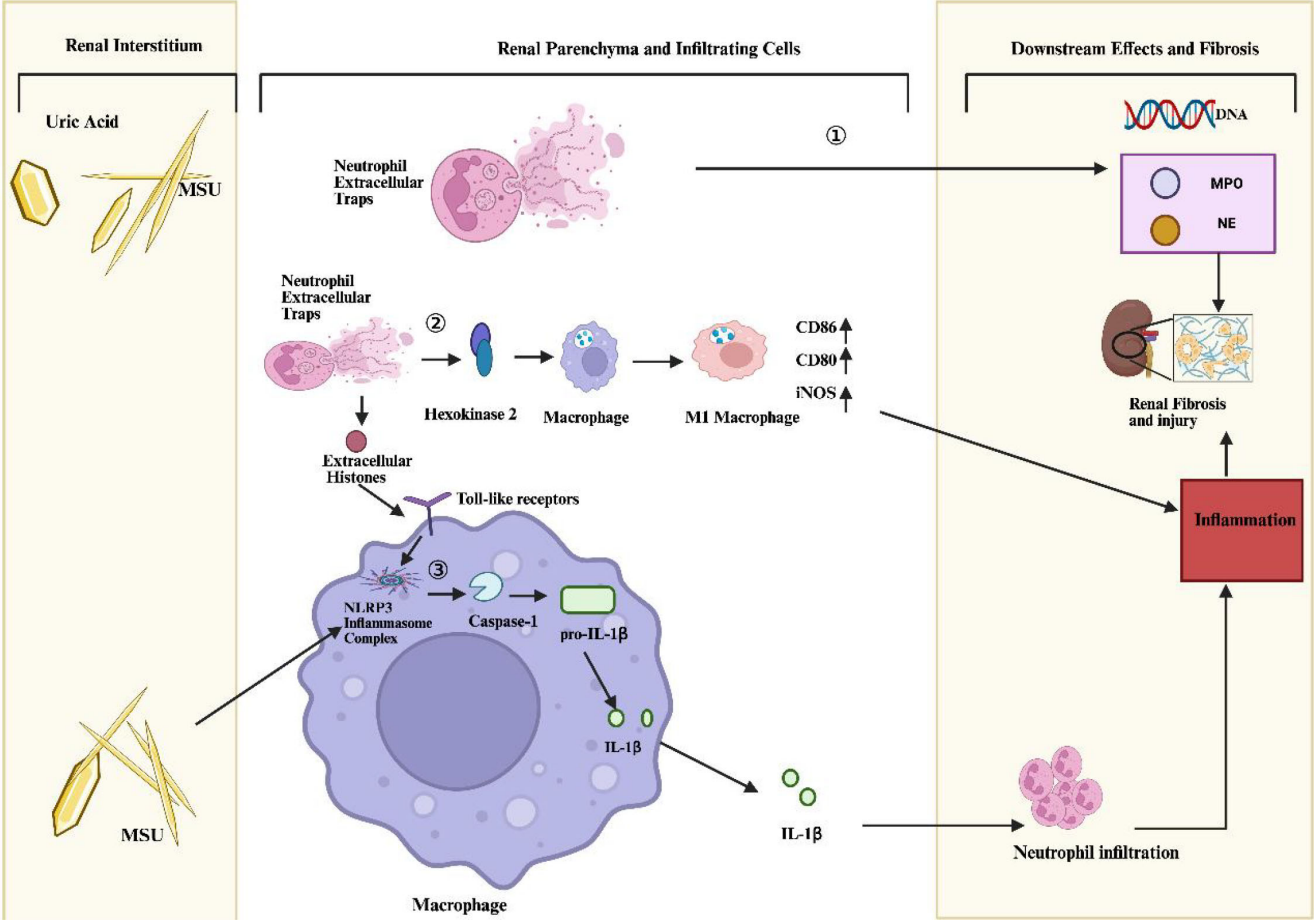
Pro-inflammatory and Injurious Effects of NETs in a Hyperuricemic. ①Environment.NETs can directly damage tissues by releasing degranulation proteins, neutrophil elastase (NE), and myeloperoxidase (MPO). ②NETs can promote the polarization of macrophages towards the M1 phenotype by targeting hexokinase-2 (HK-2), which results in the upregulated expression of CD86, CD80, and iNOS, thereby further exacerbating the local inflammatory response. ③Urate crystals also activate the NLRP3 inflammasome in macrophages, inducing caspase-1 to cleave pro-IL-1β and release mature IL-1β, thereby promoting neutrophil infiltration at inflammatory sites.

In summary, NETs likely play a multifaceted role in gouty nephropathy. Targeting NET formation could become a potential therapeutic strategy to interrupt this pathological process (**Figure 2**).

## 5 Renal injury mechanisms of NETs

### 5.1 Injury to renal vascular endothelial cells

**Figure 3:**
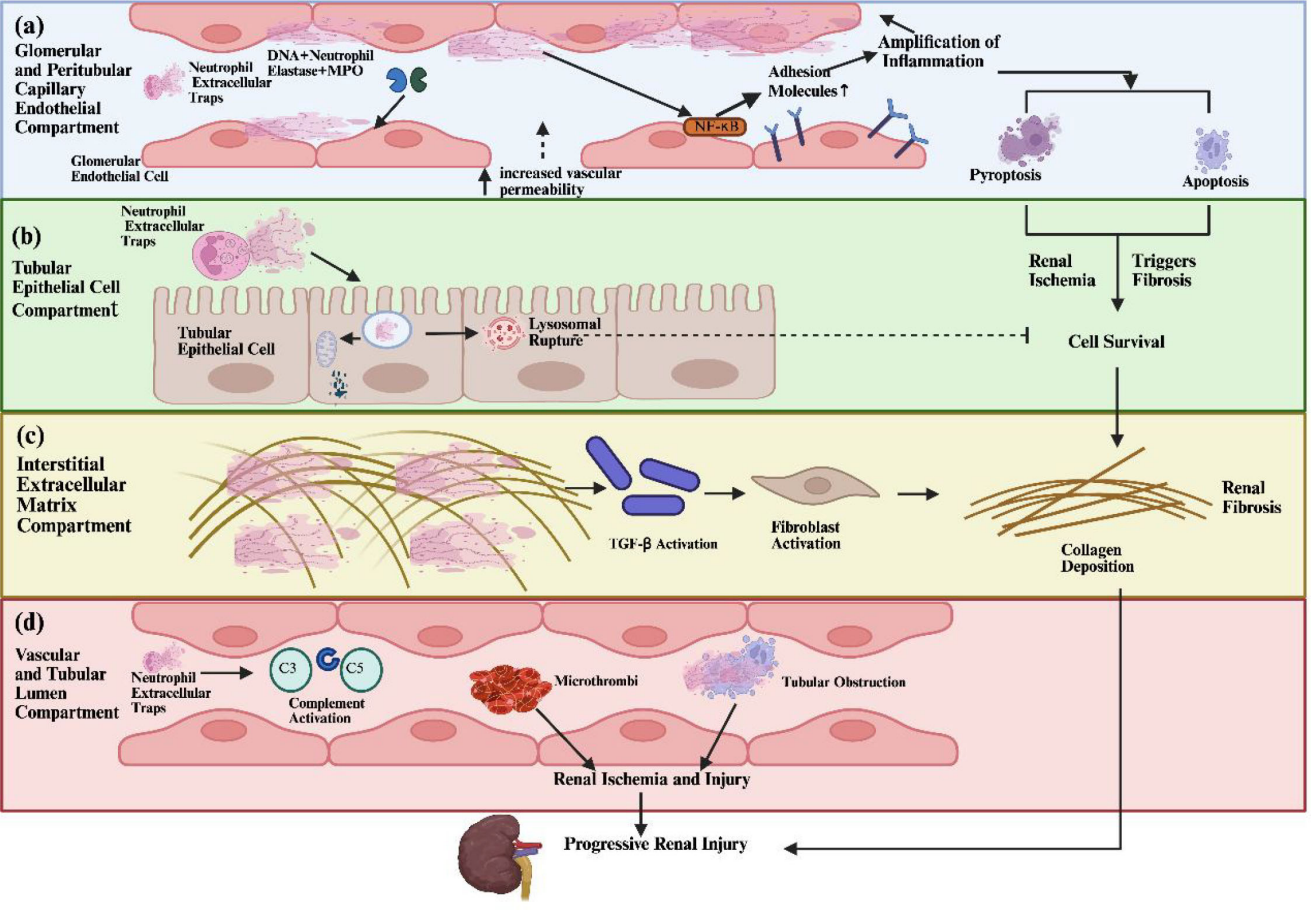
Renal Injury Mechanisms Mediated by NETs. **(a)** Various granular proteases embedded in NETs can act directly on endothelial cells, degrading intercellular junction proteins. The vascular endothelial barrier’s integrity is disrupted, leading to a significant increase in vascular permeability. **(b)** The uptake of NET components by renal tubular epithelial cells results in organelle damage. **(c)** The unique structure of NETs promotes the development of renal fibrosis. **(d)** NETs can exacerbate renal injury by activating the complement system.

The unique DNA mesh structure of NETs enables them to directly adhere to the surfaces of endothelial cells in glomerular and peritubular capillaries (6). Granular proteases embedded in NETs, such as neutrophil elastase (NE) and myeloperoxidase (MPO), can act directly on endothelial cells, degrading junctional proteins between them and damaging the basement membrane. This significantly compromises vascular barrier integrity and increases permeability (6). Studies have shown that in induced acute kidney injury models, NETs primarily aggregate around glomeruli and peritubular capillaries, and their abundance positively correlates with the degree of renal damage (65). Inhibiting NETs release or promoting NETs degradation can alleviate renal vascular endothelial injury and reverse pathological changes in the kidney, indicating a key role for NETs in mediating this damage (65). NETs and their components can act as inflammatory stimuli to activate the NF-κB pathway in endothelial cells (66). Activation of the NF-κB signaling pathway promotes the abundant expression of adhesion molecules, such as intercellular adhesion molecules and vascular adhesion molecules, by endothelial cells (67). These molecules mediate the recruitment and adhesion of circulating neutrophils, monocytes, and macrophages to the damaged vascular endothelium, allowing these cells to infiltrate the renal parenchyma by crossing the endothelial barrier. This creates a positive feedback loop: initial NETs damage endothelial cells and recruit neutrophils, and the recruited neutrophils can in turn release more NETs, thereby exacerbating inflammation and leading to the destruction of renal tissue (67). Furthermore, NETs can induce pyroptosis and apoptosis in renal vascular endothelial cells. The extensive loss of glomerular and peritubular capillary endothelial cells results in damage to functional microvasculature, affecting renal blood perfusion and oxygenation, causing local tissue ischemia and hypoxia. This compromises renal parenchymal cell function and also promotes the progression of renal fibrosis (65) (**Figure 3a**).

### 5.2 Toxic effects on renal tubular epithelial cells

The direct toxic effect of NETs on renal tubular epithelial cells is another critical mechanism mediating renal injury. The uptake of NETs or their components by renal tubular epithelial cells induces intracellular organelle damage and dysfunction. In models of acute kidney injury, the aggregation of NETs within the tubular lumen has been observed (68–70). Proximal tubular epithelial cells can reabsorb free NETs components containing various proteolytic enzymes, leading to lysosomal rupture, cellular injury, and eventual cell death (6) (**Figure 3b**).

### 5.3 Promoting the progression of renal fibrosis

The special mesh structure of NETs not only traps pathogens but can also serve as a template for extracellular matrix remodeling, promoting the deposition of collagenous fibrotic components and driving scar tissue formation (71). Neutrophil elastase on NETs can directly activate latent Transforming Growth Factor-β (TGF-β) and degrade regulatory proteins of TGF-β, thereby activating pro-fibrotic signaling pathways (71, 72). In hyperuricemic nephropathy, substantial formation and deposition of NETs can be observed in renal tissues (1) (**Figure 3c**).

### 5.4 Other injury mechanisms

NETs can exacerbate renal injury by activating the classical pathway of the complement system, leading to the formation of membrane attack complexes (5, 73). The unique fibrous mesh structure of NETs can cause physical obstruction in the kidneys, blocking the lumen of renal tubules and hindering the flow of primary urine, thereby aggravating tubular injury and renal dysfunction (74). This fibrous network can also combine with platelets and red blood cells to form microthrombi (75), occluding glomerular capillaries and leading to local renal hypoperfusion and ischemia (76, 77), which results in renal injury (**Figure 3d**).

## 6 Therapeutic strategies targeting NETs

### 6.1 Basic research on therapeutic strategies targeting NETs

With the growing understanding of the pathological role of NETs in various diseases, NET-specific inhibitory drugs have become an area of intense research interest (78–80). These inhibitors are designed to target different stages of NETs, primarily including their formation and clearance.

#### 6.1.1 Inhibition of NETs formation

In the context of gout, MSU crystals are stimuli that induce NETs formation. MSU crystals can induce NETs formation by activating the Ras-Raf-ERK signaling pathway, a process dependent on ROS production (81). PAD4 is a core enzyme in NETosis (82, 83). PAD4 inhibitors can specifically suppress histone citrullination, thereby hindering NETs formation (64, 84–88). In addition to directly inhibiting the terminal steps of NETosis, inhibiting upstream signaling initiators is equally important. ROS is a core initiating signal for NETosis, and NADPH oxidase is a major source of ROS generation. In models of MSU induced NET formation, NADPH oxidase inhibitors, such as apocynin, can effectively downregulate ROS levels and inflammation-related indicators (81). Therefore, reducing ROS to inhibit NETosis can alleviate gouty inflammation, which provides insights for targeting NETs in the treatment of gouty nephropathy. Furthermore, neutrophil elastase is another important component of NETs, positioning elastase inhibitors as a significant therapeutic target (89). Although these inhibitors are currently applied in non-gouty nephropathy models, they provide a potential reference for research on targeting NETs in gouty nephropathy treatment. Current research shows that the elastase inhibitor sivelestat can alleviate inflammation and tissue damage in lung tissue by blocking NET formation and reducing the destruction of key structural proteins in the basement membrane and extracellular matrix (57, 90, 91).In a mouse model of acute lung injury (ALI), targeted treatment of lung injury with ciclesonide encapsulated in lung-homing nanocarriers (LHN) demonstrates certain specificity (91). This therapeutic approach provides potential reference significance for targeting NETs in the treatment of gouty nephropathy (**Table 1**).

**Table 1:** Transformation table of NETs targeting strategies.

Intervention strategy	Drug	Mechanism of action	Evidence level	Kidney specificity	Potential risks	Limitations	References
Inhibition of NET formation	PAD4 inhibitor	Inhibit histone citrullination	Mainly preclinical studies; effective in multiple inflammatory models; limited in nephropathy models	Low (systemic effects)	Interfere with NETs	Lack of clinical data; insufficient targeting	(88)
	NADPH oxidase inhibitors	Inhibit ROS production	Preclinical	Low (ROS is a systemic signaling molecule)	Disrupt redox balance	Difficult to precisely inhibit pathological ROS burst	(81)
	Neutrophil elastase inhibitors	Inhibit the activity of NET-associated neutrophil elastase and interfere with NET formation	Indirect evidence; limited in gouty nephropathy models	Moderate (local administration feasible in acute lung injury mouse models, providing potential reference for NET-targeted therapy of gouty nephropathy)	May impair pathogen clearance	Evidence mainly derived from non-renal organs; requires further validation in gouty nephropathy	(81, 89–91)
Inhibition of NET formation	DNase I	Degrade the DNA backbone of NETs	Preclinical	Moderate (enzyme preparation; can be designed as kidney-targeted delivery system)	Degradation of NETs may release toxic proteins and trigger inflammation	Short half-life *in vivo*	(94, 95)
Neutralization of NET toxic components	Anti-citrullinated histone antibodies, heparin	Neutralize the cytotoxicity of free histones	Preclinical	Low (systemic administration neutralizes circulating histones)	Antibody drugs may induce immune reactions; heparin may cause bleeding tendency	Limited research in gouty nephropathy models	(102)

#### 6.1.2 Promotion of NETs clearance

Once NETs form and accumulate in tissues, clearing them can reduce their cytotoxicity and pro-inflammatory effects (92, 93). Deoxyribonuclease I (DNase I) can effectively degrade the DNA backbone of NETs, dismantling the mesh structure (10, 57, 94–97). However, DNase I has a short half-life *in vivo* and may have insufficient local concentration in the kidneys. In addition to enzymatic degradation, the phagocytosis of NETs by macrophages is also crucial. Enhancing this process can promote the timely clearance of NETs, preventing continuous damage to surrounding tissues by components such as histones (92, 98). Research has found that in rheumatoid arthritis, NETs can inhibit the osteogenic capacity of mesenchymal stem cells by activating the NF-κB pathway, while also promoting IL-8 secretion to recruit additional neutrophils, thereby perpetuating a vicious cycle (99). This indicates that the interaction between NETs and immune cells modulates their clearance. Therefore, promoting effective phagocytosis of NETs by macrophages can both clear harmful substances and shift the inflammatory microenvironment toward a reparative state, providing a potential research avenue for the treatment of gout-related renal injury (**Table 1**).

#### 6.1.3 Neutralization of the toxic components of NETs

Histones, the main protein components of NETs, exhibit potent cytotoxic and pro-inflammatory activity once released into the extracellular environment. Therefore, blocking histone release can mitigate these detrimental effects (100, 101). The administration of anti-histone antibodies or heparin can neutralize the toxicity of free histones (102). Studies have shown that a therapeutic anti-citrullinated histone antibody can inhibit NETs formation and promote their clearance (103). In acute kidney injury, NETs and their components can amplify the inflammatory response by damaging renal tubular epithelial cells and endothelial cells (6). These studies provide a potential theoretical basis for developing specific protein inhibitors to neutralize the toxicity of NETs (**Table 1**).

### 6.2 Limitations of existing research, challenges in clinical translation, and the clinical significance of NETs diagnostic biomarkers for managing gouty nephropathy patients

#### 6.2.1 Limitations and gaps in existing research

Although targeted therapy against NETs offers potential intervention strategies across the aforementioned three aspects, basic research on targeting NETs for treating gouty nephropathy remains limited by specific gaps. First, regarding the treatment of gouty nephropathy, there is a lack of efficacy data for strategies targeting neutrophil extracellular traps (NETs), such as DNase I mediated degradation or the use of PAD4 and neutrophil elastase inhibitors. Second, most existing studies are based on models of other diseases. For example, in animal models of vasculitis, using DNase I and PAD4 inhibitors can exert therapeutic effects by limiting NETs formation (104). However, as these findings are confined to animal studies, it is important to note that the characteristics, biological functions, and clearance mechanisms of NETs may vary depending on the inducing stimulus (105). Therefore, for NETs induced by MSU crystals in gouty nephropathy, the intervention effects might be different. Additionally, a bottleneck in current translational research for gouty nephropathy is the lack of ideal animal models that recapitulate the pathological features of NETs seen in human disease. Although NETs are confirmed to regulate fibrosis in multiple organ diseases (106), suitable *in vivo* models are currently lacking to analyze how NETs drive the transition from acute inflammation to chronic fibrosis specifically in the context of gout-related renal injury. Thus, it is crucial to develop composite renal injury models that closely mimic NET-associated pathology while simulating persistent hyperuricemia and intrarenal MSU crystal deposition.

#### 6.2.2 Challenges in clinical translation

Due to the complexity of NETosis, a combinatoratorial strategy using NETs inhibitors targeting distinct mechanisms can both inhibit the formation of new NETs and promote the clearance of existing ones (107). The clinical translation of NETs inhibitors still faces many challenges. Firstly, NETs is an important component of innate immunity, playing a crucial role in defending against bacterial and fungal infections. Completely, inhibiting NETs formation may impair host defense mechanisms, increasing the patient’s susceptibility to infection. Therefore, future targeted therapies against NETs need to find a balance, specifically inhibiting pathological NETosis without increasing infection risk. Furthermore, the unique anatomical structure of the kidneys poses additional barriers. NETs inhibitors that are effective *in vitro* or in the bloodstream may have difficulty reaching effective therapeutic concentrations at specific inflammatory sites within the kidneys. Therefore, developing strategies for the targeted delivery of drugs like PAD4 inhibitors or DNase I to the damaged renal tissue remains a critical challenge.

#### 6.2.3 NETs diagnostic biomarkers and clinical significance for managing gouty nephropathy patients

Research on NETs related biomarkers provides a new pathophysiological perspective for a deeper understanding of gout and gouty nephropathy. Firstly, measuring specific NETs components in the plasma of gout patients, such as Myeloperoxidase-DNA (MPO-DNA) and Neutrophil elastase-DNA (NE-DNA) complexes, shows that their levels are positively correlated with serum uric acid concentration (108). This suggests that urate crystals are potent inducers of NETosis. Secondly, in a rat model of Hypoxic renal injury (HRI), Cell-free DNA (cf-DNA), MPO-DNA, and Citrullinated histone H3 (cit-H3) elevated in parallel with renal function injury indicators like blood urea nitrogen (BUN) and Serum creatinine (Scr), further supporting the association between NETs and worsening renal function (109). In sepsis, high levels of double-stranded DNA (dsDNA) and MPO-DNA are independently associated with an increased risk of death (110). Although these studies focus on hypoxic renal injury and sepsis, their findings hold potential relevance for identifying diagnostic biomarkers in gouty nephropathy. Additionally, in the diagnosis of gouty nephropathy and the management of patients, traditional renal function indicators (serum creatinine, glomerular filtration rate) and uric acid levels cannot effectively distinguish whether kidney damage is in an active inflammatory state or a chronic non-inflammatory state. Elevated NETs biomarkers, such as MPO-DNA and cit-H3, may indicate that kidney damage is associated with an active NETosis process, providing auxiliary evidence for the diagnosis of “inflammatory” gouty nephropathy. This will help differentiate whether kidney damage in gouty nephropathy is caused by inflammation due to urate crystal deposition and NETs, or by non-inflammatory tubular injury simply due to hyperuricemia, thereby providing clues for precise treatment. Collectively, these studies offer potential strategies for the prevention and treatment of gouty nephropathy. In gouty nephropathy, elevated baseline NETs levels may predict a future rapid decline in estimated glomerular filtration rate (eGFR), aiding in patient risk stratification.

## 7 Conclusion

The role of NETs in gouty nephropathy cannot be overlooked. Firstly, the vicious cycle formed between urate crystals and NETs is a key driver of disease progression (4, 111). Urate crystals can activate the NLRP3 inflammasome in macrophages (55, 112), thereby triggering inflammatory pathways to exacerbate NETs production. Secondly, the histone components of NETs can directly induce cytotoxicity in renal vascular endothelial cells, thereby compromising nephron structure and function (101, 113, 114). These mechanisms constitute a complex network. Therefore, the understanding of gouty nephropathy can be expanded from the traditional perspectives of crystals and inflammation to include NETs mediated inflammatory responses and tissue injury. In basic research, tools such as PAD4 inhibitors and DNase I have demonstrated potential for targeted clearance of NETs (104, 115, 116).

NETs exist in a dynamic equilibrium of damage and resolution within gouty diseases (64, 65, 112).This suggests that in gouty nephropathy, NETs likely primarily exert pro-inflammatory and pro-fibrotic damaging effects, while under specific conditions, they may exhibit certain anti-inflammatory potential, forming a dynamic balance. The final outcome of NETs’ dual effects of damage and resolution in gouty nephropathy may depend on the dynamic equilibrium of several key factors. Firstly, low-level, controlled NETs formation may primarily serve to “sequester” crystals and “resolve” early inflammation, whereas massive, uncontrolled NETs release leads to excessive accumulation of toxic components like histones and proteases, overwhelming the kidneys’ inherent clearance capacity and subsequently dominating the damaging effects (4, 117). Secondly, the timing is a crucial factor. In the early stages of inflammation, NETs can physically envelop and isolate urate crystals, limiting their direct contact with surrounding renal tissue cells and, within a certain time window, containing the spread of inflammation (63), thereby restricting the scope of injury. Furthermore, the body’s clearance capacity is equally important. The kidneys’ intrinsic clearance capacity determines the duration of NETs’ presence. In chronic kidney disease states, this clearance capacity may decline, leading to persistent NET presence, continuous immune system stimulation, and accelerated fibrosis progression (64). Consequently, the therapeutic goal could be to regulate NETs levels and activity within a “therapeutic window” where they exert crystal-clearing effects without causing excessive tissue damage.

However, there are still certain challenges in the future regarding the targeted treatment of gouty nephropathy using neutrophil extracellular traps (NETs) and achieving the therapeutic time window. Firstly, targeted and precise intervention methods for the damaged local kidney tissue are required. Secondly, the safety of NETs-targeted therapies must be carefully evaluated to avoid impairing the body’s normal anti-infection defense mechanisms.
